# Comparison of reducing sugar content, sensory traits, and fatty acids and volatile compound profiles of the *longissimus thoracis* among Korean cattle, Holsteins, and Angus steers

**DOI:** 10.5713/ajas.18.0065

**Published:** 2018-07-26

**Authors:** Min Yu Piao, Hyun Jung Lee, Hae In Yong, Seok-Hyeon Beak, Hyun Jin Kim, Cheorun Jo, Komang Gede Wiryawan, Myunggi Baik

**Affiliations:** 1 Department of Agricultural Biotechnology and Research Institute of Agriculture and Life Sciences, College of Agriculture and Life Sciences, Seoul National University, Seoul 08826, Korea; 2 Feed Research Institute, Chinese Academy of Agricultural Sciences, Beijing 100081, China; 3 Institute of Green Bio Science and Technology, Seoul National University, Pyeongchang 25354, Korea; 4 Centers for Food and Bioconvergence, Seoul National University, Seoul 08826, Korea; 5 Department of Animal Nutrition and Feed Technology, Faculty of Animal Science, Bogor Agricultural University, Bogor 16680, Indonesia

**Keywords:** Korean Cattle, *Longissimus thoracis*, Reducing Sugar Content, Sensory Trait, Fatty Acids, Volatile Compounds

## Abstract

**Objective:**

This study was performed to compare fat content, reducing sugar contents, sensory traits, and fatty acid (FA) and volatile compound profiles in *longissimus thoracis* (LT) among Korean cattle (KC), Holstein (HO), and Angus (AN) steers.

**Methods:**

Twelve LT samples (about 500 g each) of KC with an average age of 31±0.42 months, an average carcass weight of 431±12.5 kg, and a quality grade (QG) of 1+ were obtained from the joint livestock products market. Twelve LT samples of HO cattle with an average age of 24±0.54 months, an average carcass weight of 402±7.81 kg, and a QG of 2 were also obtained from the same market. Twelve LT samples of AN steers with an average age of about 20 months and a QG of choice were purchased from a beef delivery company. After slaughter, samples were kept at 4°C for 42 days and prepared for immediate analysis or stored at appropriate conditions. The chemical composition, color, pH, shear force, collagen content, reducing sugars, sensory evaluation, FA composition, and volatile compound content for each LT sample were analyzed.

**Results:**

The LT of KC had the highest (p<0.05) fat content, the highest reducing sugar content, and the highest scores in the sensory evaluation (flavor, tenderness, juiciness, and overall acceptance). All the sensory traits were positively correlated (p<0.001) with intramuscular fat and reducing sugar content. Several FAs and volatile compound profiles varied among the breeds. KC LT had the highest (p<0.05) concentrations of acetaldehyde, 3-methyl butanal, and 3-hydroxy-2-butanone, and these volatile compounds were positively correlated (p<0.05) with all the sensory traits.

**Conclusion:**

Variations in fat content and reducing sugar contents and FA and volatile compound profiles may contribute to differences in the sensory quality of LT among breeds.

## INTRODUCTION

Per capita beef consumption in South Korea has increased nearly four-fold, from 2.6 kg in 1980 to 10.8 kg in 2014; this is likely a result of increasing levels of income and the westernization of Korean eating patterns [1]. Domestic beef production in South Korea is primarily based on Korean cattle (KC), but Holstein (HO) steers are also produced [2]. The self-sufficiency rate of beef consumption in South Korea has decreased and reached as low as 37.7% in 2016 [3]. South Korea’s main beef importers in 2016 were the United States and Australia, both of which predominantly imported Angus (AN) cattle [3]. Korean consumers have historically preferred KC beef to domestic HO and imported beef because they believe that the palatability (e.g., flavor, juiciness, and tenderness) of KC beef is superior to that of HO and imported beef [4]. Limited information is available on the factors that affect the preference of Korean consumers for KC beef over other breeds.

Beef flavor, juiciness, and tenderness are key factors in evaluation of meat palatability, and these three factors in combination are known to have great influence on consumers’ overall perceptions of the taste of beef [5]. Intramuscular fat (IMF) or fat content are closely related to both beef flavor and tenderness [6]. In addition to IMF contents, the composition and content of fatty acids (FAs) are also important factors in beef palatability [7]. It has been suggested that oleic acid (C18:1n9) is positively associated with beef flavor, whereas polyunsaturated fatty acid (PUFA) may be negatively associated with beef flavor [8].

During the cooking process of meat, large quantities of volatile compounds are formed, such as aldehydes, ketones, hydrocarbons, furans, thiazoles, and pyrazines; these compounds directly affect flavor [9,10]. Volatile compounds can form via several pathways, including a Maillard reaction of amino acids or peptides with reducing sugars (e.g., glucose and ribose), thermal lipid degradation, and the interaction between Maillard reaction products and lipid oxidation products [11,12]. Of these pathways, thermal lipid oxidation is primarily involved in the formation of major volatile compounds, and FA oxidation is primarily responsible for the development of flavor [13]. Therefore, different types or amounts of FAs may influence the generation of volatile compounds, the development of aroma characteristics, and the intensity of flavor upon thermal processing.

Reducing sugars, such as glucose and ribose, have been reported to contribute to improved flavor of cooked meat by reacting with amino acids to produce many important volatile compounds via Maillard reactions [14]. Currently, little information is available on the reducing sugar content of beef and its association with sensory traits. Furthermore, limited information is available regarding differences in reducing sugar content, sensory traits, and FA and volatile compound profiles among cattle breeds. This study was performed to compare the physico-chemical characteristics, reducing sugar content, sensory traits, and FA and volatile compound profiles of the *longissimus thoracis* (LT) among KC, HO, and imported AN breeds, and to identify correlations among these parameters.

## MATERIALS AND METHODS

### Beef sample preparation

The KC steers were from 12 female cows (implanted with the semen of seven different bulls), and were raised according to conventional Korean feeding methods. The HO steers were from 12 female cows (implanted with the semen of 11 different bulls), and were also raised according to conventional Korean feeding methods. Briefly, the KC male calves were weaned at 3 months of age and were then fed roughage (70%) and concentrate (30%) until 5 months of age. The calves were castrated at 6 months of age, and steers were grown in pens using group feeding. The steers were fed concentrate and roughage separately until slaughter at around 29 months of age. During growing stage (6 to 14 months of age), steers were fed a concentrate diet (1.6% of body weight [BW]/steer) and a timothy hay (approximately 0.75% of BW/steer). During fattening stage (early fattening, 15 to 23 months of age; late fattening, 24 to 29 months of age), steers were fed a concentrate diet (approximately 1.5% of BW/steer) and rice straw (1 kg/steer). The HO male calves were weaned at two months of age, then fed starter diet (30%) and hay (70%) until four months of age, and were castrated at 5 months of age. The steers were fed concentrate and roughage separately until slaughter at around 20 months of age. During growing stage (5 to 8 months of age), steers were fed a concentrate diet (approximately 2.4% of BW/steer) and a timothy hay (approximately 0.75% of BW/steer). During fattening stage (early fattening, 9 to 13 months of age; late fattening, 14 to 20 months of age), steers were fed a concentrate diet (approximately 1.8% of BW/steer) and rice straw (1 kg/steer). Cattle were slaughtered in a local municipal slaughterhouse (Bucheon, Republic of Korea). Carcass traits were evaluated by a meat grader using the Korean carcass-grading system of the Korean Institute for Animal Products Quality Evaluation [15] at 24 h post mortem. The LT samples (about 500 g each) of KC with an average age of 31±0.42 months, an average carcass weight of 431±12.5 kg, and a quality grade (QG) of 1+ were obtained from the joint livestock products market (Bucheon, Republic of Korea) immediately after grading. The LT samples of HO cattle with an average age of 24±0.54 months, an average carcass weight of 402±7.81 kg, and a QG of 2 were also obtained from the same market. Samples were then vacuum-packaged, transported under ice to a laboratory, and stored at 4°C for 42 days until the analysis. We selected a QG of 1+ for KC beef because the largest proportion (44.4%) of KC steer beef was of this grade [16]. Similarly, a QG of 2 for HO beef was selected because the largest proportion (48.6%) of HO steer beef was of this grade. The LT samples of the AN steers (choice grade) had an average age of about 20 months; they were slaughtered on the same day as the KC and HO cattle in the United States, shipped at 4°C, and imported by a beef delivery company (A-Meat Corp., Seoul, Republic of Korea). It took approximately five weeks to obtain AN samples from USA. Aging is one of significant factors affecting meat characteristics. Thus, samples of KC and HO samples also stored 42 d to give same storage time to avoid aging variation among three breeds. The choice grade of the 12 AN LT samples was randomly purchased from approximately 100 choice beef options. We selected a choice grade because it is the most popular and abundant grade in the United States. The AN cattle were raised using a conventional beef production system in the United States. After 42 days of storage, the packages containing the LT samples of the different breeds were opened and the external fat was trimmed away. The LT samples were minced using a mini chopper (CH180; Kenwood, Shanghai, China) for 30 s. Minced LT samples from various locations were pooled and stored at −70°C for analysis of the chemical composition, and collagen, reducing sugar, FA, and volatile compound contents. Samples assessed for shear force and sensory traits were collected but not minced. Some samples were used immediately for shear force evaluation, while others were stored at −70°C for the sensory evaluation.

### Proximate composition, color, pH, shear force, and collagen content

Moisture, crude protein, and crude fat contents were analyzed according to the methods of the Association of Official Analytical Chemists (AOAC) [17]. Surface-color values (International Commission on Illumination [CIE]; L*, a*, and b* values represent lightness, redness, and yellowness, respectively) were measured using a colorimeter (CR-310; Minolta Co., Ltd., Osaka, Japan). The pH of each sample was measured as described previously [18]. The samples (1 g) used for pH measurement were homogenized with distilled water (9 mL) at Lv.6 (30,000 rpm) for 30 s (T10 Basic; Ika Works, Staufen, Germany) and centrifuged at 2,265×g for 10 min (Continent 512R; Hanil Co., Ltd., Incheon, Korea). The sample pH was measured with a pH meter (SevenGo; Mettler-Toledo, Inc., Schwerzenbach, Switzerland). The shear force (N) was measured as described previously [18] using a Warner-Bratzler shear attached to a texture analyzer (CT3 10K; Brookfield Engineering Laboratories, Middleboro, MA, USA), based on slight modifications of the method developed by Kim et al [19].

The total insoluble collagen content was measured using a spectrophotometer, as described previously [18], according to a modified method of [20].

### Reducing sugars

Sugars were extracted from LT sample (1 g) by homogenization (T10 Basic; Ika Works, Germany) with 5 mL of 80% ethanol (50°C) and were centrifuged at 2,265×g for 10 min (Continent 512; Hanil Co., Ltd., Korea). The extracts were then centrifuged (Continent 512R; Hanil Co., Ltd., Korea) at 2,265×g for 10 min. The resulting supernatants were filtered (filter paper No.1; Whatman International Ltd., Springfield Mill, Kent, England) separately into 15-mL tubes and evaporated using N_2_ gas (99.999%). Next, distilled water (2 mL) was added to each tube and vortexed to dissolve the sugars. The mixture was transferred to a 2-mL microtube and centrifuged at 18,500×g for 10 min (HM-150IV; Hanil Co., Ltd., Korea). The reducing sugar content was measured using a dinitrosalicylic (DNS) acid method, as described by the [21]. In brief, 1 mL of each extract was mixed with 2 mL of the DNS solution (0.5 g of DNS acid, 8.0 g of sodium hydroxide, and 150 g of Rochelle salt in 500 mL of distilled water) in a 15-mL test tube and heated in a water bath (90°C) for 10 min. The mixture was then cooled under running water for 5 min and the absorbance was measured at 550 nm using a spectrophotometer (X-ma 3100; Human Co., Ltd., Seoul, Korea). The reducing sugar content was calculated using a standard curve developed with glucose (Sigma Corp., St. Louis, MO, USA).

### Sensory evaluation

We used similar size (15×40×15 mm^3^) of LT samples to have a uniform cooking from outside and inside of meat flesh, and the samples were cooked. The internal temperature of the samples was monitored using a digital thermometer (YF-160A Type-K; YFE, Hsinchu City, Taiwan) that was placed in the center or each edge of the grill; the samples were removed from the electronic grill (EG-GW1700, Kitchenart, Incheon, Korea) after they reached an internal temperature of 72°C. Ten untrained panelists evaluated the cooked samples for appearance, odor, taste, flavor, tenderness, juiciness, and overall acceptability. A 9-point hedonic scale, where 9 indicated *extremely liked* and 1 *indicated extremely* disliked, was employed to evaluate all of the parameters. For the evaluation, samples were placed into randomly coded white dishes and served together with drinking water to rinse the panelists’ oral cavities, following a test of each sample. The sensory characteristics were evaluated in three independent experiments.

### Fatty acid composition

The FA composition was measured as described previously [22]. Briefly, lipids in beef samples (5 g) were extracted with chloroform/methanol (100 mL) according to the procedure of [23]. Extracted lipids were methylated with BF_3_-methanol (14%, w/w), and FA methyl esters (FAMEs) were injected into a gas chromatograph (HP 7890; Agilent Technologies, Santa Clara, CA, USA). A split inlet (split ratio, 50:1) was used to inject the samples into a capillary column (SP 2560 Capillary column; 100 m×0.25 mm×0.20 μm film thickness). The inlet temperature was 210°C. N_2_ served as the carrier gas, at a constant flow rate of 1 mL/min. The column oven temperatures were as follows: 100°C for 5 min, increased to 240°C at 4°C/min, then held at 240°C for 20 min. Individual FAMEs were identified by comparison of the relative retention times of the peaks from the samples with those of the standard mixture (37-component FAME Mix; Supelco, Bellefonte, PA, USA). The FA composition of the fat was calculated based on the peak area. Relative quantities were expressed as weight percent of total FAs.

FA content (values per 100 g of beef) is also important to measure, and was obtained by using FA percentage in LT fat and fat content in LT as previously described [22]. Individual FA content (g/100 g meat) was calculated as follows:

$$\begin{array}{l} \text{Individual FA content }(\text{g FA}/100\;\text{g meat})\\ =\text{crude fat content }(\text{g fat}/100\;\text{g meat})\\ \times \text{individual FA percentage }(\%)\times 0.953\;(\text{g FA}/\text{g fat}) \end{array}$$

### Volatile compounds

To analyze the volatile compounds, samples were minced and grilled on a hot plate (PC-420D; Corning, Corning, NY, USA) until they reached an internal temperature of 72°C. Then, the sample (10 g) was immediately transferred to individually labeled 20 mL clear glass vials (N9306078; PerkinElmer, Boston, MA, USA) and closed with a polytetrafluoroethylene septa and screw cap. One minute later, the vials were placed in the oven of a headspace sampler, and extraction of the volatile compounds of the samples was performed using a headspace autosampler. The transfer line from the headspace sampler was directly connected to the injector for the gas chromatography (GC).

A PerkinElmer 680 GC equipped with a 600T mass spectrometry (MS) detector was used to analyze the volatile compounds. The compounds were separated using a HP-PLOT Q column (Agilent, Wilmington, DE, USA; 30 m×0.53 mm×0.25-μm film thickness). The GC conditions were as follows: initial oven temperature, 35°C; held for 5 min, increased by 7°C/min to 180°C, held for 0.0 min at 180°C, and then increased by 5°C/min to 250°C and held for 21 min. The transfer line temperature was maintained at 250°C. A mass spectrometer scanned from *m/z* 30 to *m/z* 250 with a 0.2 s cycle time. The ion source was set at 250°C. Headspace was maintained at 85°C for 30 min and a ramped oven temperature was used (50°C for 3 min, increased by at 5°C/min to 240°C, and maintained for 9 min). The inlet temperature was 210°C. Helium served as the carrier gas, delivered at a constant flow rate of 20 mL/min. The resolved MS spectra obtained from the custom scripts were matched against reference mass spectra by using the National Institute of Standards and Technology (NIST) mass spectral search program for application to the NIST/US Environmental Protection Agency (EPA)/National Institutes of Health (NIH) mass spectral library (ver. 2.0). A GC chromatogram was used to quantify the volatile compounds, and MS was used to identify the volatile compounds. Results of the analyses are expressed as percentages of the total chromatographic area.

### Statistical analysis

Data (12 animals/group) were analyzed by analysis of variance using the general linear model procedure (Proc GLM) of SAS software (SAS Institute, Cary, NC, USA). The LSMEANS PDIFF option was used to compare differences among mean values at p<0.05. The CORR procedure of SAS was used to calculate Pearson’s correlation coefficients.

## RESULTS AND DISCUSSION

### Chemical composition, physico-chemical parameters, collagen and reducing sugar contents, and sensory traits

The chemical compositions, physico-chemical parameters, collagen and reducing sugar contents, and sensory traits of the LT of KC, HO, and AN breeds are shown in Table 1. The KC had the highest (p<0.05) fat content (21.1%), followed by HO (7.08%) and AN (3.14%), whereas KC had the lowest (p<0.05) moisture and crude protein contents. This is consistent with previous study that reported higher (p<0.05) crude fat content in the loin of KC versus that of imported beef [24].

The LT of both KC and AN had similar CIE a* (redness) values, but the LT of HO had a lower (p<0.05) value than that of the two other breeds (Table 1). CIE L*- (lightness) and CIE b*-value (yellowness) did not differ (p>0.05) among the three breeds. Cho et al [24] also reported that the CIE L* value of the loin did not differ among KC with a QG of 1+, KC with a QG of 1, Australian AN, and crossbred breeds (AN and Hereford mix). Imported AN had the highest (p<0.05) pH, followed by KC and HO.

The shear force of the LT was the lowest (p<0.05) in KC, followed by HO and AN (Table 1). Shear force is known to be an indicator of meat tenderness. A previous study reported that tenderness increased with a decrease in shear force [25], and that shear force in turn decreased with an increase in QG [26]. In our study, the lowest shear force was seen in KC; this may be attributable to that meat having the highest fat content or IMF content. Total collagen content in the LT did not differ (p>0.05) among the three breeds (Table 1). Soluble collagen content was highest (p<0.05) in AN, but did not differ between KC and HO. Insoluble collagen content was highest (p<0.05) in HO, and again did not differ between KC and AN.

The reducing sugar content of the LT was highest (p<0.05) in KC (0.42%), and the content did not differ between HO (0.18%) and AN (0.19%) (Table 1). Our study is the first to show that KC had the highest reducing sugar content among several breeds. Volatile flavor compounds mainly produce meat aromas and flavors during cooking. Maillard reactions between amino acids or peptides with reducing sugar are key processes in generating volatile compounds [27]. The reducing sugar for this reaction included ribose, glucose, glucose 6-phosphate, and ribose 5-phosphate [28]. It was reported that reducing sugar may act as important precursors of flavor in chicken meat [29]. Thus, the two-fold higher reducing sugar content in the LT from KC may influence the production of volatile compounds during cooking.

All sensory parameters, including flavor, tenderness, juiciness, and overall acceptance, were highest (p<0.05) in KC, and no differences in flavor or overall acceptance were observed between HO and AN (Table 1). The IMF content positively affected the sensory quality [6,18]. The IMF is strongly correlated with fat content [18]. In our study, the LT of KC had a significantly higher fat content than that of the other two breeds, and the shear force of LT from KC was the lowest among the three breeds. Collectively, a higher fat content and lower shear force may have contributed to the higher sensory quality of the LT of KC compared with the other two breeds. Additionally, the reducing sugar content was highest in KC, which may also in part contribute to the superior sensory traits of this breed, as discussed above.

### Percentages and contents of FA

Of the 18 FAs identified in this study, the percentage of C18:1n9 was the highest, ranging from 39% to 44%, followed by palmitic acid (C16:0) (26.5% to 30%) and stearic acid (C18:0) (11.4% to 15.9%) (Table 2). The percentages of C16:0, palmitoleic acid (C16:1), and C18:1n9 were lowest (p<0.01) in AN, and did not differ between KC and HO. Cho et al [24] also reported higher proportions of C16:1 and C18:1n9 in the loin fat of KC versus AN. The percentage of monounsaturated fatty acids (MUFAs) in the LT of KC and HO was higher (p<0.05) than that in AN beef. This may be because the proportion of C18:1n9 was higher in KC and HO than in AN. The MUFA deposition in the triacylglycerol fraction increased with increasing fat content, whereas PUFA was preferentially incorporated into the phospholipid fraction in the cell membranes [30]. Thus, the higher fat content of KC compared with AN may have contributed directly to the higher MUFA content of KC. The percentages of C18:0, linoleic acid (C18:2), and arachidonic acid (C20:4) were lowest (p<0.01) in KC. The PUFA percentage of LT fat was lowest (p<0.01) in KC and highest in AN. Cho et al [24] also reported lower C18:2 and PUFA percentages for the *longissimus* muscle fat of KC versus AN. In addition, Jeong et al [31] reported that the *longissimus lumborum* of KC had lower C18:2 and PUFA percentages than that of HO. Hwang and Joo [8] reported that C18:1n9 was positively associated with beef flavor, whereas PUFAs were negatively associated with beef flavor. Taken together, variation in the proportions of these FAs may in part have contributed to differences in the sensory traits of the LT of different breeds.

Using the FA percentage and fat content of the LT, individual FA contents (g FA/100 g LT) were calculated based on a conversion factor (0.953; [32]). Among all FAs, the C18:1n9 content of the LT was the highest, ranging from 1.9 to 8.33 g/100 g meat, followed by C16:0 (1.27 to 5.62 g/100 g meat) and C18:0 (0.76 to 2.11 g/100 g meat) (Supplementary Table S1). The content of most FAs in the LT, including C16:0, C16:1, C18:0, and C18:1n9, were highest (p<0.001) in KC and lowest (p<0.001) in AN. These results are likely attributable to the fat content in the LT being highest in KC and lowest in AN.

### Volatile compounds

A total of 31 volatile compounds, including aldehydes, hydrocarbons, ketones, pyrazines, alcohols, and terpenes, were detected in the LT of the three cattle breeds (Table 3). The abundant volatile compounds, accounting for 7.28% to 60% of the total volatile compounds, included acetaldehyde and 2-propanone. The moderate compounds, accounting for 1.26% to 25.9% of the total volatile compounds, included 2-methyl butanal, hexanal, and methanethiol. The minor compounds, accounting for 0.01% to 8.5% of the total volatile compounds, included propanal, butanal, 3-methyl butanal, pentanal, heptanal, benzaldehyde, octanal, nonanal, butane, carbon disulfide, 2-butene, n-pentane, hexane, heptane, octane, ethylacetate, 2-butanone, 2,3-butanedione, 3-hydroxy-2-butanone, 2-heptanone, 2,3-octanedione, dimethyl sulfide, ethanol, isopropyl alcohol, 1-pentanol, and 1,3,5-cycloheptatriene.

The percentages of produced aldehydes varied among the three breeds. Acetaldehyde and 3-methyl butanal contents were highest (p<0.05) in KC, followed by HO and AN (Table 3). The 2-methyl butanal content was similar between KC and HO, and was lowest in AN. In contrast, propanal, pentanal, hexanal, heptanal, and octanal contents were highest (p<0.05) in AN, and similar between KC and HO. During the formation of volatile compounds, Strecker degradation is one of the most important reactions related to the generation of the precursors of thermal formation aromas. The Strecker aldehydes acetaldehyde (sharp, penetrating, fruity), 2-methyl propanal (penetrating, green), 3-methyl butanal (malty, green), and 2-methyl butanal (ethereal, bitter, almond, green), which are well-known volatile components of cooked beef, may be derived from alanine, valine, leucine, and isoleucine, respectively [33]. Of these aldehydes, acetaldehyde is associated with many reactions that may explain the formation of meaty aromas: 3,5-dimethyl-1,2,4-trithiolane, trithioacetaldehyde, thialdine, 2,4-dimethylthiazole, 2,4-dimethyl-5-ethylthiazole, and 2,4,5- trimethyl-3-thiazoline together appear to impart a complex meat flavor during cooking. Acetaldehyde provides the carbon skeleton on their structures [33]. In addition, cysteine is also a well-known precursor of meat flavor. Some compounds, including mercaptoacetaldehyde, hydrogen sulfide, and acetaldehyde, were identified as common products when cysteine was boiled with various carbonyl compounds. Of these compounds, acetaldehyde is produced by Strecker degradation or cysteine hydrolysis [34]. Acetaldehyde was the most abundant volatile compound detected in this study. Among the three cattle breeds, the acetaldehyde content was significantly higher in KC than in HO and AN, and there was a positive correlation between acetaldehyde content and sensory scores. The reducing sugar content was also significantly higher in KC, which may in part be due to the relatively high content of sugars in this type of beef; reducing sugar is a substrate in Maillard reactions or complex Maillard and Strecker reactions [35]. Thus, higher percentages of acetaldehydes may in part have contributed to the higher sensory scores of KC beef.

Hydrocarbon percentages varied among the three breeds. The methanethiol percentage in the LT was highest (p<0.01) in HO, and lowest in AN. Butane, hexane, and ethylacetate were present in similar proportions in HO and AN, while KC had a lower butane percentage (p<0.05) than that of the other two breeds. The percentages of n-pentane and heptane were highest (p<0.01) in AN, while the KC and HO breeds had similar percentages of these compounds. Carbon disulfide and octane percentages did not differ (p>0.05) among the three breeds. Hydrocarbons are derived from lipid thermal oxidation, which is a reaction catalyzed by heme compounds such as myoglobin and hemoglobin [36]. Although hydrocarbon components make up a certain proportion of volatile compounds, Min et al [37] reported that both saturated and unsaturated hydrocarbons play a minor role in the flavor of roast beef, as they contain relatively weak, non-beef-like odors. In this study, the correlations between flavor and the percentages of most hydrocarbons, including butane, carbon disulfide, 2-butene, n-pentane, hexane, heptane, octane, and ethylacetate were not significant (data not shown). Collectively, hydrocarbon variation among the three breeds did not seem to significantly affect the flavor of LT.

Among the ketones, 2-propanone percentages were similar in both KC and HO LT, and lowest (p<0.01) in AN LT. The percentage of 2-butanone was highest (p<0.01) in HO LT, and lowest in AN LT. Both 2,3-butanedione and 3-hydroxy-2-butanone percentages were highest (p<0.01) in KC, while HO and AN had similar percentages of these compounds. Both 2-heptanone and 2,3-octanedione percentages were highest (p<0.01) in AN, while KC and HO had similar contents of these compounds. Previous reports have shown that 2,3-butanedione and 3-hydroxy-2-butanone contents in AN were higher in prime grades compared with choice or standard grades [38]. Ketones are also related to fat content, and ketone content increases with increasing fat content [39].

### Correlation

We analyzed Pearson correlation coefficients for the chemical composition, physical-chemical parameters, and sensory traits of pooled LTs from KC, HO, and AN (Supplementary Table S2). Fat content was one of the factors most strongly associated with sensory traits (flavor, tenderness, juiciness, and overall acceptance); these results were consistent with our previous study that showed positive correlations between fat content and sensory traits (juiciness and overall acceptance) [18].

Little information about the correlation between beef carcass characteristics and reducing sugar content is available. In this study, we analyzed Pearson correlation coefficients for chemical composition and sensory traits with the reducing sugar content of pooled LTs from the three breeds. The reducing sugar content in the LT was positively correlated (0.63≤r≤0.71; p<0.001) with fat content and sensory traits (flavor and overall acceptance) (Table 4). We also found that KC LT had the highest reducing sugar content and the best sensory traits among the three breeds. As mentioned above, reducing sugar, especially glucose, can contribute to an improved meat flavor by reacting with amino acids to produce important volatile compounds via Maillard reactions [35]. Our study thus implies that higher reducing sugar content may positively affect flavor and overall acceptance.

Crude fat content was positively correlated with C14:0, C16:0 (0.48≤r≤0.50, p<0.01), C16:1, C18:1n9, and MUFA percentages (0.53≤r≤0.73, p<0.001), and negatively correlated with C18:0, C18:2, and PUFA percentages (−0.75≤r≤ −0.71, p<0.001), in the LT fat (Supplementary Table S3). This is generally consistent with our previous study [22] that showed positive correlations of C18:1n9 and MUFA percentages, and negative correlations of C18:2 and PUFA percentages, with LT fat content.

Sensory traits, including flavor, tenderness, and juiciness, were positively correlated (0.35≤r≤0.61, p<0.05) with C16:0, C18:1n9, and MUFA percentages in LT fat, and negatively correlated (−0.70≤r≤ −0.42, p<0.05) with C18:2 and PUFA percentages (Table 5). Mandell et al [40] reported that C18:1n9 might be positively associated with beef flavor, whereas PUFA might be negatively associated with beef flavor. Other studies have also shown that C18:1n9 is positively associated with beef flavor, whereas C18:2 is negatively associated with beef flavor [41]. O’Quinn [42] reported that MUFAs, such as lauric acid (C12:1), myristic acid (C14:1), C16:1, and C18:1n9, were all positively correlated with overall flavor desirability (beefy, browned/grilled, brothy, buttery, and sweet flavors), and negatively correlated with bloody/metallic, grassy, gamey, livery, fishy, and sour flavor intensities, which can impart bad flavor to the beef. Collectively, C18:1n9, and MUFA percentages may positively affect sensory traits, whereas C18:2 and PUFA percentages may negatively affect sensory traits. In this study, we found that C18:1n9 and MUFA percentages were highest, and C18:2 and PUFA percentages were lowest, in KC LT fat among the three breeds. Taken together, the high percentages of C18:1n9 and MUFA, and low percentages of C18:2 and n-6 PUFA, in KC LT fat may contribute to the sensory traits of KC beef being rated as the best for Korean consumers.

We analyzed the correlation coefficients for chemical composition, physico-chemical parameters, reducing sugar content, and sensory traits with the volatile compound content of pooled LTs from KC, HO, and AN. Both the crude fat (0.63≤r≤0.70, p<0.001) and reducing sugar contents (0.39≤r≤0.57, p<0.05) were positively correlated with several volatile compounds, including acetaldehyde, 3-methyl butanal, 2,3-butanedione, and 3-hydroxy-2-butanone (Table 6). The percentages of acetaldehyde, 3-methyl butanal, 2,3-butanedione, and 3-hydroxy-2-butanone were also positively correlated (0.39≤r≤0.66, p< 0.05) with flavor and/or overall acceptance. Our results are consistent with previous studies that reported positive correlations between the 3-methyl butanal contents of loins from AN, HO, and American Wagyu with buttery/beef fat flavor [43]. Both 2-methyl butanal and 3-methyl butanal have been associated with nutty and buttery odors, which positively affect flavor [44]. Other studies have also reported that concentrations of 2,3-butanedione and 3-hydroxy-2-butanone had positive correlations with beef flavor [38,43]. Both 2,3-butanedione and 3-hydroxy-2-butanone are widely used to produce an artificial buttery flavor, and they are also known to impart a buttery flavor to beef [45]. We also found that crude fat and reducing sugar contents as well as the percentages of 3-methyl butanal, 2,3-butanedione, and 3-hydroxy-2-butanone, were highest in KC LT among the three breeds, which may in part contribute to high levels of volatile compounds in KC beef. This in turn may play a positive role in the superior flavor of KC beef versus the other two breeds for Korean consumers. The impact of 2,3-butanedione and 3-hydroxy-2-butanone on beef flavor in this study may have been minor because these compounds were present in relatively low amounts (less than 1.0% of all volatile compounds). Further study is warranted to elucidate the role of reducing sugar in the formation of volatile compounds, and their contribution to beef flavor.

In contrast, both crude fat (−0.71≤r≤ −0.60, p<0.001) and reducing sugar contents (−0.49≤r≤ −0.39, p<0.05) were negatively correlated with several volatile compounds, including propanal, pentanal, hexanal, heptanal, octanal, and n-pentane (Table 6). None of these volatile compounds showed significant correlations with flavor and overall acceptance, although they were negatively correlated with tenderness and juiciness.

We analyzed the correlation coefficients for FA percentages (in LT fat) with volatile compounds. The percentages of C16:0, C16:1, C18:1n9, and MUFA of LT fat were positively correlated (0.41≤r≤0.78, p<0.05) with acetaldehyde, 2-methyl butanal, 3-methyl butanal, and 2-propanone percentages (Table 7). The percentages of C18:1n9 and MUFA of LT fat were also positively correlated (0.49≤r≤0.61, p<0.01) with 2,3-butanedione and 3-hydroxy-2-butanone, which impart a buttery scent to beef. Both C18:1n9, and MUFA are known to be positively associated with beef flavor [32]. Currently, limited information is available on the generation of volatile compounds from specific FAs. In contrast, the C16:0, C16:1, C18:1n9, and MUFA percentages of LT fat were negatively correlated (−0.81≤r≤ −0.50, p<0.01) with several volatile compounds, including propanol, pentanal, hexanal, and n-pentane (Table 7). Pentanal and hexanal can impart pungent, fishy, and grassy odors, which are negatively associated with flavor [46]. Our study showed that these compounds were negatively correlated with tenderness and juiciness, and not correlated with flavor or overall acceptance (Table 6).

The percentages of C18:0, C18:2, and PUFA of LT fat were negatively correlated (−0.74≤r≤ −0.38, p<0.05) with acetaldehyde, 2-methyl butanal, 3-methyl butanal, 2-propanone, 2,3- butanedione, and 3-hydroxy-2-butanone percentages, whereas all of these FAs were positively correlated (0.60≤r≤0.83, p< 0.001) with propanal, pentanal, hexanal, butane, and n-pentane (Table 7). C18:0 is negatively correlated with beef fat aroma and flavor [8]. Also, PUFAs, including C18:2, are known to be oxidized easily, so they can easily exert a negative effect on beef flavor [47].

Overall, our results indicate that specific FAs can be positively or negatively correlated with volatile compounds. Our data imply that FA composition may in part affect the sensory traits of beef, including flavor, through the generation of volatile compounds.

In our study, both KC and HO were raised in Korea with a KC feed regimen, while AN were raised in the United States with a typical US cattle feed regimen. These dietary and environmental differences may affect beef characteristics. Therefore, our study has limitation for interpretation of results.

In conclusion, the fat content, reducing sugar content, and sensory traits of the LT varied among KC, HO, and AN steers. The KC LT had the highest fat content and reducing sugar contents, and the best sensory traits (flavor, tenderness, juiciness, and overall acceptance). The fat content and reducing sugar contents were positively correlated with all of the sensory traits, suggesting that these factors may positively affect beef flavor. C16:0, C18:1n9, and MUFA may positively affect sensory traits, whereas C18:2 and PUFA may negatively affect sensory traits. The percentages of different volatile compounds in the LT also varied among the three breeds. The KC had the highest percentage of volatile compounds, including acetaldehyde, 3-methyl butanal, and 3-hydroxy-2-butanone, and these compounds were positively correlated with flavor. Our results demonstrated that variations in fat content, reducing sugar content, and FA and volatile compound profiles may contribute to differences in the sensory characteristics of the LT among breeds. The results of this study enhance our understanding of the association of reducing sugar and volatile compound contents with the sensory traits of beef. This information may help in determining beef palatability. This study has limitations of interpretation, considering that beef samples were collected in cattle with different conditions including feeding method, diet type, slaughter weight, and slaughter age and that these factors may affect IMF contents and beef quality and palatability attributes [48].

## Supplementary Data



## Figures and Tables

**Table 1:** Chemical composition, physico-chemical parameter, collagen and reducing sugar contents, and sensory traits in the *longissimus thoracis* from Korean cattle (KC), Holstein (HO), and Angus (AN) steers (n = 12)

Item	KC	HO	AN	SEM	p-value
Chemical composition					
Moisture (%)	57.6^c^	67.4^b^	73.7^a^	0.99	0.001
Crude protein (%)	18.2^b^	22.1^a^	21.5^a^	0.28	0.001
Crude fat (%)	21.1^a^	7.08^b^	3.14^c^	1.14	0.001
Physico-chemical parameter					
CIE L^*^	45.8	45.3	45.4	0.27	0.82
CIE a^*^	17.0^a^	16.1^b^	17.5^a^	0.18	0.01
CIE b^*^	3.72	3.00	3.35	0.16	0.27
pH	5.43^b^	5.19^c^	5.71^a^	0.04	0.001
Shear force (N)	24.3^c^	32.0^b^	39.6^a^	1.73	0.001
Collagen content					
Total (mg/g)	4.64	5.18	5.03	0.15	0.19
Soluble (mg/g)	1.92^b^	1.91^b^	2.46^a^	0.11	0.03
Insoluble (mg/g)	2.72^b^	3.27^a^	2.57^b^	0.11	0.01
Reducing sugar (%)	0.42^a^	0.18^b^	0.19^b^	0.02	0.001
Sensory traits1)					
Appearance	5.90^a^	5.70^a^	5.33^b^	0.07	0.004
Odor	6.23^a^	4.70^b^	5.23^b^	0.14	0.001
Taste	6.80^a^	3.90^b^	4.47^b^	0.25	0.001
Flavor	6.77^a^	4.03^b^	4.60^b^	0.23	0.001
Tenderness	6.73^a^	5.23^b^	3.90^c^	0.22	0.001
Juiciness	6.03^a^	4.70^b^	3.97^c^	0.18	0.001
Overall acceptance	6.83^a^	4.23^b^	4.73^b^	0.23	0.001

SEM, standard error of the mean; CIE, International Commission on Illumination.

1) The score was evaluated with 10 semi-trained panelists (1, extremely dislike; 5, neither dislike nor like; 9, extremely like).

a–c Means with different letters within the same row differ (p<0.05).

**Table 2:** Fatty acid composition (% of fat) in the *longissimus thoracis* from Korean cattle (KC), Holstein (HO), and Angus (AN) steers (n = 12)

Fatty acid	KC	HO	AN	SEM	p-value
C12:0	0.11^a^	0.11^a^	0.00^b^	0.01	0.001
C13:0	0.04	0.06	0.10	0.01	0.14
C14:0	4.09^a^	3.90^a^	3.02^b^	0.14	0.004
C14:1	1.39^a^	1.36^a^	0.68^b^	0.09	0.001
C15:0	0.23^c^	0.33^b^	0.50^a^	0.02	0.001
C16:0	30.0^a^	29.3^a^	26.5^b^	0.36	0.001
C16:1	5.12^a^	5.01^a^	3.39^b^	0.19	0.001
C17:0	0.36^c^	0.92^b^	1.94^a^	0.15	0.001
C17:1	0.50^c^	0.66^b^	1.12^a^	0.05	0.001
C18:0	11.4^b^	12.4^b^	15.9^a^	0.42	0.001
C18:1t	0.22^b^	0.22^b^	0.42^a^	0.03	0.002
C18:1n9	44.0^a^	41.6^a^	39.0^b^	0.54	0.001
C18:2	1.60^b^	2.55^b^	5.20^a^	0.31	0.001
C18:3	0.04^b^	0.09^b^	0.19^a^	0.01	0.001
C18:2_9c11t_+C18:2_10t12c_	0.36	0.31	0.37	0.02	0.54
C20:1	0.27^a^	0.18^b^	0.22^ab^	0.01	0.02
C20:3	0.14^b^	0.30^a^	0.36^a^	0.03	0.001
C20:4	0.17^c^	0.72^b^	1.04^a^	0.08	0.001
SFA1)	46.2	47.0	48.0	0.40	0.24
USFA2)	53.8	53.0	52.0	0.40	0.25
MUFA3)	51.5^a^	49.1^a^	44.9^b^	0.64	0.001
PUFA4)	2.31^c^	3.97^b^	7.16^a^	0.42	0.001

SEM, standard error of the mean; SFA, saturated fatty acids; USFA, unsaturated fatty acids; MUFA, monounsaturated fatty acids; PUFA, polyunsaturated fatty acids.

1) SFA = C12:0+C13:0+C14:0+C15:0+C16:0+C17:0+C18:0.

2) USFA = C14:1+C16:1+C17:1+C18:1t+C18:1c+C20:1.

3) MUFA = C14:1+C16:1+C17:1+C18:1t+C18:1c+C20:1.

4) PUFA = C18:2+C18:3+C20:3+C20:4+C18:2_9c11t_+C18:2_10t12c_.

a–c Means with different letters within the same row differ (p<0.05).

**Table 3:** Volatile compounds (%) in the *longissimus thoracis* from Korean cattle (KC), Holstein (HO), and Angus (AN) steers (n = 12)

Volatile compound	KC	HO	AN	SEM	p-value
Aldehyde					
Acetaldehyde	60.0^a^	49.1^b^	31.5^c^	2.43	0.001
Propanal	0.29^b^	0.53^b^	2.48^a^	0.19	0.001
Butanal	0.06^b^	1.17^a^	0.33^ab^	0.22	0.1
2-Methyl butanal	2.79^a^	2.76^a^	1.26^b^	0.13	0.001
3-Methyl butanal	1.25^a^	0.98^b^	0.54^c^	0.06	0.001
Pentanal	0.81^b^	1.56^b^	8.50^a^	0.66	0.001
Hexanal	1.76^b^	3.81^b^	25.9^a^	2.07	0.001
Heptanal	0.07^b^	0.14^b^	0.92^a^	0.08	0.001
Benzaldehyde	0.24^a^	0.29^a^	0.17^b^	0.01	0.004
Octanal	0.05^b^	0.09^b^	0.58^a^	0.04	0.001
Nonanal	0.02	0.01	0.01	0.00	0.79
Hydrocarbon					
Methanethiol	8.89^b^	12.8^a^	3.98^c^	0.75	0.001
Butane	0.94^b^	1.08^a^	1.12^a^	0.03	0.02
Carbon disulfide	1.77	2.01	0.96	0.20	0.13
2-Butene	0.29^b^	0.36^a^	0.30^b^	0.01	0.02
n-Pentane	0.73^b^	1.70^b^	6.47^a^	0.49	0.001
Hexane	0.41^b^	0.83^a^	0.65^ab^	0.06	0.01
Heptane	0.05^b^	0.06^b^	0.53^a^	0.04	0.001
Octane	0.45	1.56	1.30	0.48	0.63
Ethylacetate	0.32^b^	1.27^a^	0.62^ab^	0.17	0.1
Ketone					
2-Propanone	14.8^a^	13.9^a^	7.28^b^	0.68	0.001
2-Butanone	0.96^b^	1.49^a^	0.38^c^	0.11	0.001
2,3-Butanedione	0.08^a^	0.04^b^	0.03^b^	0.00	0.001
3-Hydroxy-2-butanone	0.89^a^	0.14^b^	0.17^b^	0.08	0.001
2-Heptanone	0.04^b^	0.05^b^	0.24^a^	0.02	0.001
2,3-Octanedione	0.05^b^	0.14^b^	1.01^a^	0.09	0.001
Pyrazine					
Dimethyl sulfide	0.48	0.53	0.65	0.05	0.29
Alcohol					
Ethanol	1.07	0.88	0.45	0.18	0.35
Isopropyl alcohol	0.26^b^	0.49^a^	0.24^b^	0.03	0.001
1-Pentanol	0.06^b^	0.08^b^	0.72^a^	0.06	0.001
Terpene					
1,3,5-Cycloheptatriene	0.18^b^	0.22^b^	0.66^a^	0.05	0.001

SEM, standard error of the mean.

a–c Means with different letters within the same row differ (p<0.05).

**Table 4:** Pearson correlation coefficients of chemical composition and sensory traits with reducing sugar contents of pooled *longissimus thoracis* from Korean cattle (KC), Holstein (HO), and Angus (AN) steers (n = 36)

Item	Reducing sugar content
Chemical composition	
Moisture	−0.70***
Crude protein	−0.72***
Crude fat	0.71***
Sensory traits	
Flavor	0.63***
Tenderness	0.59***
Juiciness	0.55***
Overall acceptance	0.63***

*** p<0.001.

**Table 5:** Pearson correlation coefficients of sensory traits with fatty acid composition (% of fat) of pooled *longissimus thoracis* from Korean cattle (KC), Holstein (HO), and Angus (AN) steers (n = 36)

Item	C14:0	C16:0	C16:1	C18:0	C18:1n9	C18:2	SFA	USFA	MUFA	PUFA
Flavor	0.24	0.35*	0.21	−0.30	0.35*	−0.42*	−0.07	0.07	0.35*	−0.46**
Tenderness	0.43**	0.61***	0.49**	−0.58***	0.53***	−0.70***	−0.18	0.18	0.57***	−0.70***
Juiciness	0.32	0.50**	0.37*	−0.50**	0.51**	−0.59***	−0.21	0.20	0.52***	−0.60***
Overall acceptance	0.26	0.37*	0.24	−0.30	0.32	−0.43**	−0.03	0.03	0.33	−0.47**

SFA, saturated fatty acids; USFA, unsaturated fatty acids; MUFA, monounsaturated fatty acids; PUFA, polyunsaturated fatty acids.

* p<0.05;

** p<0.01;

*** p<0.001.

**Table 6:** Pearson correlation coefficients of chemical composition, physico-chemical parameter, reducing sugar, and sensory traits with volatile compounds of pooled *longissimus thoracis* from Korean cattle (KC), Holstein (HO), and Angus (AN) steers (n = 36)1)

Item	Acetaldehyde	Propanal	2-Methyl butanal	3-Methyl butanal	Pentanal	Hexanal	Heptanal	Octanal	Methanethiol	n-Pentane	2-Propanone	2-Butanone	2,3-Butanedione	3-Hydroxy-2-butanone
Chemical composition														
Moisture	−0.73***	0.68***	−0.68***	−0.73***	0.72***	0.72***	0.66***	0.74***	−0.38*	0.75***	−0.72***	−0.26	−0.69***	−0.67***
Crude protein	−0.50**	0.39*	−0.35*	−0.43**	0.42*	0.39*	0.37*	0.40*	−0.04	0.46**	−0.44**	0.08	−0.56***	−0.59***
Crude fat	0.70***	−0.62***	0.65***	0.69***	−0.67***	−0.67***	−0.60***	−0.68***	0.35*	−0.71***	0.68***	0.20	0.67***	0.63***
Physico-chemical parameters														
pH	−0.47**	0.63***	−0.77***	−0.50**	0.67***	0.69***	0.64***	0.71***	−0.84***	0.63***	−0.56***	−0.69***	−0.22	0.05
Shear force	−0.43**	0.52***	−0.43**	−0.60***	0.46**	0.42*	0.47**	0.49**	−0.21	0.43**	−0.37*	−0.23	−0.26	−0.36*
Reducing sugar	0.52**	−0.40*	0.36*	0.56***	−0.44**	−0.42*	−0.39*	−0.40*	−0.01	−0.49**	0.40*	−0.04	0.39*	0.57***
Sensory traits														
Flavor	0.40*	−0.20	0.21	0.52***	−0.23	−0.26	−0.17	−0.27	−0.12	−0.29	0.28	−0.07	0.54***	0.66***
Tenderness	0.67***	−0.61***	0.64***	0.79***	−0.64***	−0.67***	−0.57***	−0.68***	0.34*	−0.66***	0.64***	0.31	0.56***	0.58***
Juiciness	0.58***	−0.54***	0.55***	0.72***	−0.57***	−0.60***	−0.49**	−0.57***	0.28	−0.59***	0.57***	0.29	0.62***	0.62***
Overall acceptance	0.39*	−0.22	0.20	0.52***	−0.25	−0.28	−0.19	−0.28	−0.13	−0.31	0.31	−0.03	0.52***	0.66***

1) Correlation coefficient values of several volatile compounds (butanal, butane, carbon disulfide, octane, ethylacetate, benzaldehyde, nonanal, 2-butene, hexane, heptane, 2-heptanone, 2,3-octanedione, dimethyl sulfide, ethanol, isopropyl alcohol, 1-pentanol, 1,3,5-cycloheptatriene) were r<0.50 or p>0.05, and these data are not shown in this table.

* p<0.05;

** p<0.01;

*** p<0.001.

**Table 7:** Pearson correlation coefficients between fatty acid composition (% of fat) and volatile compounds of pooled *longissimus thoracis* from Korean cattle (KC), Holstein (HO), and Angus (AN) steers (n = 36)1)

Item	Acetaldehyde	Propanal	2-Methyl butanal	3-Methyl butanal	Pentanal	Hexanal	Methanethiol	Butane	n-Pentane	2-Propanone	2,3-Butanedione	3-Hydroxy-2-butanone
C14:0	0.39*	−0.43**	0.50**	0.47**	−0.46**	−0.47**	0.43**	−0.19	−0.49**	0.46**	0.21	0.15
C16:0	0.41*	−0.52***	0.59***	0.71***	−0.50**	−0.50**	0.43**	−0.17	−0.51**	0.45**	0.28	0.24
C16:1	0.58***	−0.60***	0.65***	0.48**	−0.66***	−0.66***	0.56***	−0.35*	−0.68***	0.65***	0.29	0.21
C18:0	−0.68***	0.69***	−0.74***	−0.57***	0.74***	0.74***	−0.60***	0.37*	0.76***	−0.71***	−0.52***	−0.38*
C18:1n9	0.73***	−0.59***	0.58***	0.42**	−0.69***	−0.70***	0.33*	−0.52***	−0.71***	0.64***	0.61***	0.50**
C18:2	−0.72***	0.63***	−0.72***	−0.68***	0.77***	0.79***	−0.48**	0.62***	0.83***	−0.74***	−0.52***	−0.43**
SFA	−0.48**	0.45**	−0.36*	−0.06	0.47**	0.45**	−0.29	0.24	0.44**	−0.44**	−0.42*	−0.30
USFA	0.48**	−0.44**	0.36*	0.06	−0.46**	−0.45**	0.29	−0.23	−0.43**	0.44**	0.41*	0.30
MUFA	0.78***	−0.67***	0.68***	0.47**	−0.78***	−0.78***	0.46**	−0.56***	−0.81***	0.75***	0.61***	0.49**
PUFA	−0.73***	0.60***	−0.70***	−0.67***	0.75***	0.77***	−0.43**	0.63***	0.82***	−0.72***	−0.54***	−0.46**

SFA, saturated fatty acids; USFA, unsaturated fatty acids; MUFA, monounsaturated fatty acids; PUFA, polyunsaturated fatty acids.

1) Correlation coefficient values of several volatile compounds (butanal, carbon disulfide, octane, ethylacetate, 2-butanone, heptanal, benzaldehyde, octanal, nonanal, 2-butene, hexane, heptane, 2-heptanone, 2,3-octanedione, dimethyl sulfide, ethanol, isopropyl alcohol, 1-pentanol, 1,3,5-cycloheptatriene) were r<0.50 or p>0.05, and these data are not shown in this table.

* p<0.05;

** p<0.01;

*** p<0.001.
