# Supplementary material for: Comparison of reducing sugar content, sensory traits, and fatty acids and volatile compound profiles of the longissimus thoracis among Korean cattle, Holsteins, and Angus steers
Source: Asian-Australas J Anim Sci. 2018 Jul 26;32(1):126–36. doi: 10.5713/ajas.18.0065 (PMC6325407; doi:10.5713/ajas.18.0065)
Supplement: Supplementary file 1 [file ajas-18-0065-supplmentary.pdf]

Supplementary Table 1. Fatty acid contents (g/100g meat)[1)] in the *longissimus thoracis* from Korean cattle (KC), Holstein (HO), and Angus (AN) steers (n=12)

**Fatty acid**	**KC**	**HO**	**AN**	**SEM**	**p-value**
C12:0	0.02^{a}	0.01^{b}	0.00^{c}	0.00	0.001
C13:0	0.01	0.01	0.01	0.00	0.61
C14:0	0.77^{a}	0.34^{b}	0.15^{c}	0.05	0.001
C14:1	0.27^{a}	0.12^{b}	0.03^{c}	0.02	0.001
C15:0	0.04^{a}	0.03^{b}	0.02^{b}	0.00	0.001
C16:0	5.62^{a}	2.51^{b}	1.27^{c}	0.33	0.001
C16:1	0.97^{a}	0.43^{b}	0.17^{c}	0.06	0.001
C17:0	0.06	0.08	0.10	0.01	0.31
C17:1	0.09^{a}	0.06^{b}	0.06^{b}	0.00	0.001
C18:0	2.11^{a}	1.05^{b}	0.76^{c}	0.11	0.001
C18:1t	0.04^{a}	0.02^{b}	0.02^{b}	0.00	0.005
C18:1n9	8.33^{a}	3.55^{b}	1.90^{c}	0.51	0.001
C18:2	0.30^{a}	0.22^{b}	0.23^{b}	0.01	0.003
C18:3	0.01	0.01	0.01	0.00	0.35
C18:2$_{9c11t}$ + C18:2$_{10t12c}$	0.07^{a}	0.03^{b}	0.02^{b}	0.01	0.001
C20:1	0.05^{a}	0.02^{b}	0.01^{b}	0.00	0.001
C20:3	0.03^{a}	0.03^{a}	0.02^{b}	0.00	0.01
C20:4	0.03^{c}	0.06^{a}	0.04^{b}	0.00	0.001
SFA[2)]	8.63^{a}	4.02^{b}	2.30^{c}	0.48	0.001
USFA[3)]	10.2^{a}	4.53^{b}	2.50^{c}	0.61	0.001
MUFA[4)]	9.75^{a}	4.19^{b}	2.18^{c}	0.59	0.001
PUFA[5)]	0.43^{a}	0.34^{b}	0.31^{b}	0.02	0.002

SEM, standard error of the mean.

[1)] Individual fatty acid content per meat (g/100g meat) was calculated as described in Materials and Methods.

[2)] SFA (saturated fatty acids) = C12:0 + C13:0 + C14:0 + C15:0 + C16:0 + C17:0 + C18:0.

[3)] USFA (unsaturated fatty acids) = C14:1 + C16:1 + C17:1 + C18:1t + C18:1c + C20:1.

[4)] MUFA (monounsaturated fatty acids) = C14:1 + C16:1 + C17:1 + C18:1t + C18:1c + C20:1.

[5)] PUFA (polyunsaturated fatty acids) = C18:2 + C18:3 + C20:3 + C20:4 + C18:2$_{9c11t}$ + C18:2$_{10t12c}$.

$^{a-c}$ Means with different letters within the same row differ ($p < 0.05$).

Supplementary Table 2. Pearson correlation coefficients among chemical composition, physico-chemical parameters, and sensory traits of pooled *longissimus thoracis* from Korean cattle (KC), Holstein (HO), and Angus (AN) steers (n=36)

**Item**	**Crude protein**	**Crude fat**	**CIE *L****	**CIE *a****	**CIE *b****	**pH**	**Shear force**	**Flavor**	**Tenderness**	**Juiciness**	**Overall acceptance**
Chemical composition											
Moisture	0.82***	-0.98***	-0.09	0.10	-0.24	0.08	0.53***	-0.71***	-0.82***	-0.79***	-0.68***
Crude protein	–	-0.88***	-0.04	-0.05	-0.14	-0.14	0.32	-0.71***	-0.54***	-0.55***	-0.67***
Crude fat		–	0.05	-0.05	0.23	-0.08	-0.48**	0.71***	0.76***	0.73***	0.67***
Physico-chemical parameters											
CIE *L**			–	0.25	0.49**	0.26	-0.03	0.14	0.13	0.08	0.14
CIE *a**				–	0.75***	0.30	0.07	0.30	-0.01	0.06	0.29
CIE *b**					–	0.16	-0.03	0.41*	0.34*	0.38*	0.40*
pH						–	0.14	0.23	0.03	0.12	0.26
Shear force							–	-0.27	-0.45**	-0.39*	-0.30
Sensory traits											
Flavor								–	0.80***	0.84***	0.99***
Tenderness									–	0.96***	0.81***
Juiciness										–	0.85***

* $p < 0.05$; ** $p < 0.01$; *** $p < 0.001$.

Supplementary Table 3. Pearson correlation coefficients of chemical composition and physico-chemical parameters with fatty acid composition (% of fat) of pooled *longissimus thoracis* from Korean cattle (KC), Holstein (HO), and Angus (AN) steers (n=36)

**Item**	**C14:0**	**C16:0**	**C16:1**	**C18:0**	**C18:1n9**	**C18:2**	**SFA**	**USFA**	**MUFA**	**PUFA**
Chemical composition										
Moisture	-0.49**	-0.55***	-0.56***	0.73***	-0.70***	0.77***	0.39*	-0.39*	-0.76***	0.79***
Crude protein	-0.32	-0.28	-0.35*	0.50**	-0.56***	0.51***	0.36*	-0.35*	-0.59***	0.56***
Crude fat	0.48**	0.50**	0.53***	-0.71***	0.67***	-0.72***	-0.39*	0.38*	0.73***	-0.75***
Physico-chemical parameters										
pH	-0.52***	-0.56***	-0.61***	0.66***	-0.28	0.56***	0.16	-0.16	-0.43**	0.51**
Shear force	-0.49**	-0.63***	-0.47**	0.45**	-0.12	0.47**	-0.10	0.11	-0.23	0.46**

* $p < 0.05$; ** $p < 0.01$; *** $p < 0.001$.
